# Lyme Borreliosis in Finland, 1995–2014

**DOI:** 10.3201/eid2308.161273

**Published:** 2017-08

**Authors:** Eeva Sajanti, Mikko Virtanen, Otto Helve, Markku Kuusi, Outi Lyytikäinen, Jukka Hytönen, Jussi Sane

**Affiliations:** University of Turku, Turku, Finland (E. Sajanti, J. Hytönen);; National Institute for Health and Welfare, Helsinki, Finland (M. Virtanen, O. Helve, M. Kuusi, O. Lyytikäinen, J. Sane)

**Keywords:** Lyme borreliosis, erythema migrans, epidemiology, Finland, register-based study, vector-borne infections, tick-borne, Borrelia burgdorferi, Ixodes, ticks, bacteria

## Abstract

We investigated the epidemiology of Lyme borreliosis (LB) in Finland for the period 1995–2014 by using data from 3 different healthcare registers. We reviewed data on disseminated LB cases from the National Infectious Diseases Register (21,051 cases) and the National Hospital Discharge Register (10,402 cases) and data on primary LB (erythema migrans) cases from the Register for Primary Health Care Visits (11,793 cases). Incidence of microbiologically confirmed disseminated LB cases increased from 7/100,000 population in 1995 to 31/100,000 in 2014. Incidence of primary LB cases increased from 44/100,000 in 2011 to 61/100,000 in 2014. Overall, cases occurred predominantly in women, and we observed a bimodal age distribution in all 3 registers. Our results clearly demonstrate that the geographic distribution of LB has expanded in Finland and underscore the importance of LB as an increasing public health concern in Finland and in northern Europe in general.

Lyme borreliosis (LB) is an infectious disease caused by the spirochete *Borrelia burgdorferi* sensu lato and transmitted by the *Ixodes* spp. ticks. It is characterized by multiple signs and symptoms, varying from the early phase erythema migrans (EM) to neurologic manifestations, arthritis, and acrodermatitis chronica atrophicans and less often to cardiac conduction disorders (*1*). In the United States, ≈30,000 new LB cases are reported annually to the Centers for Disease Control and Prevention, although current estimates suggest the total number of cases to be 10-fold higher (*2*–*4*). In Europe, the annual number of LB cases exceeds 85,000, according to estimates by the World Health Organization (*4*), and high incidences have been reported from several countries and regions (*5*–*10*). The incidence of LB has increased over the past decades in several countries in Europe, the United States, and Canada (*4*,*5*,*8*,*11*–*14*). This change might reflect the increased abundance and expanded geographic distribution of *Ixodes* ticks to new habitats (*15*–*17*) but also increased awareness of the infection among healthcare providers and the general population.

LB is a notifiable infectious disease in only some countries in Europe, and the reporting practices and surveillance methods and definitions vary widely. Because of absent or inadequate national surveillance systems for LB observations (*8*), most epidemiologic data are derived from studies performed on populations with increased risk or in LB-endemic regions (*6*,*18*,*19*). In Finland, the epidemiology of LB was previously investigated in 1988 (*20*), but increased abundance and northward distribution of *Ixodes* spp. ticks in northern Europe (including Finland) have been more recently reported (*15*,*21*,*22*).

Finland has well-established health registers in place, maintained by the National Institute for Health and Welfare (NIHW), facilitating population-based epidemiologic analyses of infectious diseases. Research results based on the register data of Finland are likely to reflect the epidemiologic situation of LB in northern Europe. In this study, we investigated the incidence and geographic distribution of clinically diagnosed LB (i.e., erythema migrans [EM]) for the period 2011–2014 and those of microbiologically confirmed LB for the period 1995–2014, covering the whole of Finland. 

## Methods

### Study Population

In Finland (population 5.5 million), the national healthcare system is organized into 20 geographically and administratively defined hospital districts (HDs) (Technical Appendix Figure 1). The autonomous region of the Åland Islands is considered a 21st HD. The estimated population in HDs ranges from 28,700 to 1.6 million. Sixteen HDs have primary- and secondary-care hospitals, and 5 HDs also provide tertiary-care services.

### Data Sources

To analyze the demographic characteristics, seasonality, and geographic distribution of LB, we reviewed data from the National Infectious Diseases Register (NIDR), National Hospital Discharge Register (Hilmo), and the Register for Primary Health Care Visits (Avohilmo). All 3 registers are maintained by NIHW.

### NIDR for Microbiologically Confirmed LB Cases

Routine surveillance of LB in Finland is laboratory based. Since 1995, microbiologic laboratories performing LB diagnostics in Finland notify NIDR electronically of any positive findings (i.e., serologic or molecular confirmation). Each notification includes the following information: specimen date, each patient’s unique national identity code, date of birth, sex, and place of residence. For this study, we extracted all microbiologically confirmed LB cases primarily representing disseminated LB infections from NIDR that were reported during 1995–2014. Multiple notifications for the same LB case received within a 3-month period were combined as 1 case.

### Avohilmo and Hilmo Registers for Outpatient and Inpatient Healthcare Visits

According to national guidelines in Finland, when a typical EM is observed after a possible tick exposure, it is diagnosed clinically without any laboratory testing by general practitioners in the primary healthcare setting. Since 2011, these outpatient healthcare visits from the primary healthcare units (municipal health centers and health center wards) have been registered under Avohilmo. These cases are not reported to NIDR because the laboratory diagnosis is missing. Notifications in Avohilmo include the patient’s national identity code, age, sex, place of healthcare service, information concerning healthcare admission, investigations, treatment, and discharge diagnoses according to the International Classification of Diseases, 10th Revision (ICD-10). LB cases in Avohilmo were defined as illnesses diagnosed with the ICD-10 code A69.2 (“Lyme borreliosis”). Only the first discharge of each patient was included to avoid recurrent visits with the same diagnosis code being analyzed multiple times in the study. We used Avohilmo data to estimate the number of clinically diagnosed LB cases and simultaneously improve the estimate of the total number of LB cases in Finland during 2011–2014.

Another register, Hilmo, contains nationwide linkable data on all inpatient hospital discharges during 1996–2014 and is comparable to Avohilmo by the notification information; however, LB cases registered under Hilmo for the most part represent disseminated disease. We used Hilmo data to determine the proportions of different clinical manifestations of disseminated LB cases. We have provided detailed descriptions of LB case definitions, diagnostic practices, and laboratory methods used in routine diagnostics (online Technical Appendix Methods).

### Statistical Methods

We calculated the crude estimation of the total number and incidence of LB in Finland by summing clinically diagnosed (Avohilmo) and microbiologically confirmed cases (NIDR) together on the basis of 2 assumptions. First, the number of cases of EM diagnosed clinically in the primary healthcare setting does not substantially overlap with the microbiologically confirmed cases representing disseminated LB cases, as validated by individual-level register-linkage studies (J. Sane, unpub. data). Second, on average, 70% of all LB diagnoses are appropriately coded with the ICD-10 code A69.2 in Avohilmo by the general practitioners (M. Virtanen, unpub. data).

In the time trend analysis by HDs, we considered microbiologically confirmed LB cases in NIDR and outpatient LB cases in Avohilmo. We used Poisson regression for the trend analyses, and statistical significance was defined as p<0.01. We performed the analyses with Stata version 14.0 (StataCorp LLP, College Station, TX, USA). We calculated the average annual incidences of the microbiologically confirmed LB cases over 4 different periods: 1995–1999, 2000–2004, 2005–2009, and 2010–2014. We used data from the National Population Information System as denominators to calculate annual incidence rates and to calculate age- and sex-specific average annualized incidence rates.

## Results

### Demographic Characteristics of LB Case-Patients

**Figure 1:**
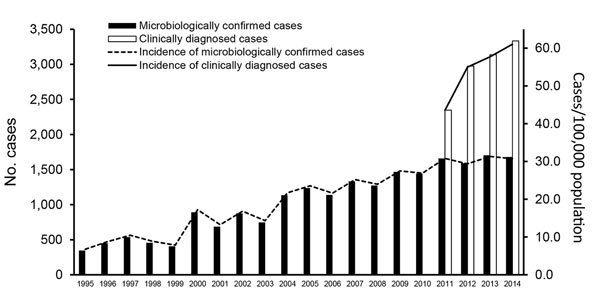
Number and incidence of microbiologically confirmed Lyme borreliosis cases reported in the National Infectious Diseases Register during 1995–2014 and clinically diagnosed cases reported in the Register for Primary Health Care Visits during 2011–2014, Finland.

We identified a total of 21,051 microbiologically confirmed LB cases in NIDR (Figure 1). The number of LB cases increased ≈5-fold, from 345 (7/100,000 population) in 1995 to 1,679 (31/100,000) in 2014. On average, ≈3,000 clinically diagnosed LB cases were identified annually in Avohilmo, yielding a total of 11,793 cases. The annual incidence increased from 44/100,000 population in 2011 to 61/100,000 in 2014 (Figure 1). We estimated the total number of annual LB cases to be 5,011 cases in 2011 (incidence of 93/100,000 population) and 6,440 cases in 2014 (118/100,000 population).

**Figure 2:**
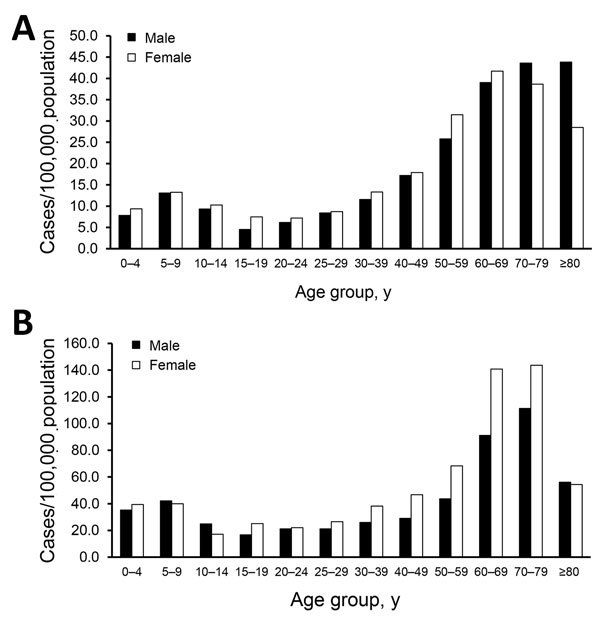
Incidence rates of microbiologically confirmed Lyme borreliosis cases reported in the National Infectious Diseases Register during 1995–2014 (A) and clinically diagnosed cases reported in the Register for Primary Health Care Visits during 2011–2014 (B), by age and sex of case-patients, Finland.

Most (54.0%) microbiologically confirmed cases occurred in women (n = 11,373). We observed a bimodal age distribution, with high incidence rates occurring in the age group of 5–9 years in both sexes, after which incidence again increased from the age of 40 years, peaking in the age group of 60–69 years among women and >70 years in men (Figure 2, panel A). We did not observe any other significant sex-specific differences in incidences across age groups.

Out of the clinically diagnosed cases, 59.7% occurred in women (n = 7,042). Again the age distribution was bimodal, but the second peak occurring in the age groups 60–79 years was notably discernble (Figure 2, panel B). In these age groups, LB incidence was distinctively higher among women than among men (on average 142/100,000 among women and 111/100,000 among men). Case-patients were predominantly female across the age groups except in children 5–14 years of age and persons >80 years of age.

Regarding hospital discharges, we identified a total of 10,402 cases with an LB diagnosis (56.2% of case-patients were female); incidence increased from 0.4/100,000 population in 1996 to 19/100,000 in 2014. We have provided a detailed description of characteristics of case-patients and the different clinical manifestations of disseminated LB (Technical Appendix Results).

### Geographic Distribution and Time Trend

**Figure 3:**
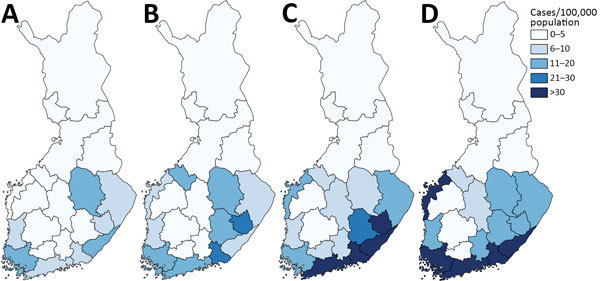
Incidence rates of microbiologically confirmed Lyme borreliosis cases, by hospital district and period, Finland, 1995–2014. A) 1995–1999; B) 2000–2004; C) 2005–2009; D) 2010–2014. The Åland Islands are not shown; only the hospital districts on the mainland are shown.

In most of the HDs (15/21 [71.4%]), incidence of microbiologically confirmed LB cases increased significantly over time (Figure 3). We observed the most notable increasing trend in western, southern, and southeastern Finland. The highest average annual incidences in the past 5 years (2010–2014) were reported in southeastern Finland, with HD-specific rates of 49–57 cases/100,000 population (Technical Appendix Table 1, Figure 1). The lowest incidences of LB were reported in northern and northeastern Finland, with 1–4 cases/100,000 population. The Åland Islands is a hyperendemic region for LB with the average annual incidence of 1,597/100,000 population during 2010–2014, with an increasing trend (p<0.01). The average annual incidence of the whole country (average of all HDs) during 2010–2014 was 30/100,000 population.

We observed the highest average annual incidences of clinically diagnosed LB during 2011–2014 in eastern and southeastern Finland, with HD-specific rates of 143–162 cases/100,000 population, and in southwestern Finland (83/100,000 population). The average annual incidence in the Åland Islands was 885/100,000 population. The countrywide average annual incidence during 2011–2014 was 54/100,000 population. Even during the period of 4 years, incidence increased significantly (p<0.05) in 8 HDs, most notably in eastern and southern Finland (Technical Appendix Table 2, Figure 1).

### Seasonality

**Figure 4:**
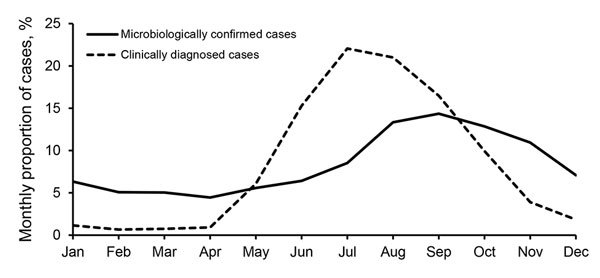
Monthly distribution of microbiologically confirmed Lyme borreliosis cases reported in the National Infectious Diseases Register during 1995–2014 and clinically diagnosed cases reported in the Register for Primary Health Care Visits during 2011–2014, Finland.

Microbiologically confirmed LB cases were reported throughout each year, although we observed a pronounced peak in seasonality in September (14.4% of all LB cases during 1995–2014) (Figure 4). More than 50% of LB cases were reported during August–November. For clinically diagnosed LB cases, seasonality was even more pronounced, and only a few cases were reported during the wintertime in Finland (November–April). Most cases (≈75%) occurred during June–September, with the peak in July (22.1% of all LB cases) and August (21.0%). Year-to-year variation in seasonality was minor among microbiologically confirmed and clinically diagnosed cases (data not shown).

## Discussion

By using 3 nationwide registers, we examined the changes in the incidence and geographic distribution of LB in Finland during a ≈20-year period. Our data allowed us to analyze the incidence of microbiologically confirmed disseminated LB cases and clinically diagnosed LB infections (reflecting EM). The incidence of LB in Finland increased significantly from 1995 to 2014, reflecting the epidemiologic situation of LB in northern Europe. As of 2014, ≈1,700 microbiologically confirmed LB cases are diagnosed yearly, compared with a few hundred cases just 15–20 years ago. When clinically diagnosed EM cases are also considered, the crude estimate of the total LB incidence reached 120/100,000 population in 2014. In 2015, after our study period, ≈1,900 microbiologically confirmed LB cases and 3,514 clinically diagnosed cases were recorded, further confirming the increasing trend (https://sampo.thl.fi/pivot/prod/fi/ttr/shp/fact_shp?row=area-12260&column=time-12059&filter=reportgroup-12465).

The increasing trend in LB incidence has been reported in several countries in Europe, various US states, and Canada in the past decade (*4*). Multiple studies suggest that milder winter temperatures, humid summers, and extended spring and autumn seasons attributable to climate change might enable the tick vectors to spread to higher altitudes and latitudes and thereby increase the risk for tickborne infections in northern Europe, including Finland (*5*,*16*,*17*). As a part of our study, a questionnaire concerning the current and previous laboratory methodology was sent to all 8 microbiological laboratories performing LB diagnostics in Finland to assess whether changes in the diagnostic methods could explain the increased incidence of microbiologically confirmed LB cases. The results indicated that no such changes can be identified. If anything, the methods have become more specific through the adoption of a 2-tier approach to *Borrelia* serologic testing.

Substantial variation exists in the regional incidences of LB throughout Finland. The Åland Islands, which is a hyperendemic region both for ticks and LB, stands out from the other HDs. Outside the Åland Islands, the highest incidences are concentrated in the coastal areas of Finland, especially along the coastline of the Baltic Sea and the southwestern archipelago. Southwestern Finland, including the archipelago, has long been well-known as a region with high tick abundance (*10*). During 1995–2000, the LB incidence was highest in southwestern Finland, but ≈15 years later, the southeastern areas of Finland have surpassed the southwestern areas in terms of LB incidence. In general, in almost all HDs in the southern half of Finland, LB incidence increased during 1995–2014. Central-western parts of Finland remain low-incidence regions. The same regions can be identified as areas of low tick density on a map of the geographic distribution of ticks in Finland in 2015 (*23*).

We observed a bimodal age-specific distribution in microbiologically confirmed LB cases and clinically diagnosed LB cases. The similar 2-peaked age distribution has been noted in other studies of LB in the United States and in Europe, but unlike in the previous studies, the second peak in our study occurred in older age groups in both sexes (*6*,*11*,*19*,*24*,*25*). One explanation for the peak in older age groups could be increased levels of leisure activity; in Finland, certain outdoor activities, such as berry picking and gardening, might be more popular among older persons, which might increase their exposure to tick bites. Furthermore, because of the aging immune system, the elderly might be at an increased risk for disseminated LB, which is observed as an increased number of LB cases, especially in NIDR (*26*).

The preponderance of women and girls with cases of EM has been reported in various other epidemiologic studies in Europe, and our data on clinically diagnosed LB cases (59.7% of which occurred in women and girls) are in accordance with those previous reports (*7*,*27*–*30*). Incidence of clinically diagnosed LB was higher only among boys 5–14 years of age and men >80 years of age. In adults, especially in the 50–79-year age group, incidence was distinctively higher among women. In terms of microbiologically confirmed LB, differences in LB incidence between men and women were not as large, but women were still slightly predominant across all age groups. However, in the >70-year age group, men were clearly overrepresented. In France, the proportion of men that were hospitalized because of LB during 2004–2012 was substantially higher than the proportion of women (57.8% vs. 42.2%), whereas women represented 52% of the LB cases reported by general practitioners (*19*). The immunologic or biologic mechanisms that might explain why older men would be more likely to have disseminated LB than women are unknown (*30*). Women might tend to notice EM more often or they might seek the healthcare services more actively while still in the EM phase of the infection. Either way, in our study, a preponderance of men with microbiologically confirmed LB cases was only observed in the older age groups.

The seasonal distribution of clinically diagnosed LB cases peaked in July and August, followed by a 1–2-month incubation period, before disseminated LB peaked in September. Both the host-seeking tick activity and the human exposure to ticks attributable to summertime outdoor activities affect the seasonality of LB. According to our data, the EM stage infections are observed during the warm summer months during June–September, and few EM cases are registered in Avohilmo during November–April. The incubation period for disseminated LB ranges from weeks to months, which results in microbiologically confirmed LB cases being reported to NIDR throughout the year, although to a lesser extent during the winter season in Finland (November–April), when only chronic manifestations of LB typically are reported.

The proportions of Lyme neuroborreliosis (9.3%) and Lyme arthritis (4.3%) cases identified were surprisingly low compared with the total number of case-patients discharged from the hospitals during the study period. A reasonable explanation might involve the reporting accuracy; most LB cases are likely registered only under the ICD-10 code A69.2 (“Lyme borreliosis”) instead of the more specific codes referring to Lyme neuroborreliosis and Lyme arthritis. However, the completeness and accuracy of Hilmo data in general have been found to be on a high level (*31*).

We acknowledge some limitations in this study. First, Avohilmo does not yet cover occupational and private healthcare visits. Thus, the number of clinically diagnosed cases is underestimated in this study, particularly among working-age persons. Second, the reliability of correctly diagnosed EM cases is highly dependent on the healthcare professionals reporting the cases to Avohilmo. However, EM is well-recognized among healthcare workers in Finland, and every healthcare visit must be registered with an ICD-10 code. We have adjusted our total estimate on the number of clinically diagnosed LB cases with a correction factor reflecting the known inaccuracies in reporting. However, the trend in LB incidence in Avohilmo is similar to that of microbiologically confirmed LB in NIDR during the past few years. Third, the awareness of LB among healthcare professionals and the public might differ geographically and seasonally, which might cause differences in diagnostic activity. Also, the location where the infection occurred might be different than the place of residence, and the fact that NIDR does not contain any clinical information is a limitation. To further evaluate the register data and to refine our incidence estimates, register-linkage studies on an individual level are needed and ongoing, including the assessment of personalized disease and antibiotic prescription patterns. Risk factors for LB should also be comprehensively assessed. We plan to expand routine surveillance of LB to also include clinically diagnosed cases and will further validate the use of primary healthcare visits for surveillance purposes.

In this study, we showed an increase in the incidence of LB in Finland during a ≈20-year period and described the changes that have taken place in the geographic distribution of LB. The epidemiologic data of LB are useful for healthcare professionals, the general public, and the media to highlight the areas and seasons of the highest infection risk. Furthermore, the results of this study stress the importance of LB as an increasing public health concern and are valuable to the public health decision-makers in guiding surveillance and intervention strategies (e.g., vaccine development), as well as increasing the awareness of the disease among the public.

Technical Appendix Additional description of methods and results in a study of Lyme borreliosis in Finland, 1995–2014.
