# Supplementary material for: Lyme Borreliosis in Finland, 1995–2014
Source: Emerg Infect Dis. 2017 Aug;23(8):1282–8. doi: 10.3201/eid2308.161273 (PMC5547811; doi:10.3201/eid2308.161273)
Supplement: Technical Appendix — Additional description of methods and results in a study of Lyme borreliosis in Finland, 1995–2014. [file 16-1273-Techapp-s1.pdf]

Article DOI: https://doi.org/10.3201/eid2308.161273

Lyme Borreliosis in Finland, 1995–2014

Technical Appendix

Methods

Case Definitions for Lyme Borreliosis (LB), Lyme Neuroborreliosis (LNB), and Lyme Arthritis (LA)

Cases in Avohilmo reflect clinically diagnosed EM cases. EM can be diagnosed as a reddish/blueish skin rash often with a central clearing expanding at least up to 5 cm in diameter in several days to weeks after a tick bite. No laboratory testing is required since the rapid expansion of the rash makes it distinctive from other skin lesion (*1*). Moreover, borrelia serology is usually negative at the early phase of infection (*2*).

Microbiologically confirmed LB cases are diagnosed by the detection of borrelia-specific IgG and/or IgM antibodies in serum and/or cerebrospinal fluid (CSF), and subsequently notified to the NIDR. In Finland, serology is based on the two-tier testing where borrelia-specific antibodies are detected by a sensitive enzyme immuno assay (EIA) followed by a more specific immunoblot. In total, eight laboratories (including both public and private units) perform LB laboratory diagnostics.

We defined a LNB case as a patient diagnosed with the following ICD-10 codes: "A69.2" (Lyme borreliosis) and either "G01.9" (meningitis) and/or "G63.0" (polyneuropathy). A case of LA was defined as a patient diagnosed with "A69.2" and "M01" (arthritis in Lyme disease). LNB is clinically diagnosed as lymphocytic meningitis, radiculoneuritis, or cranial neuritis with the laboratory confirmation of the central nervous system involvement which includes CSF pleocytosis and detection of intrathecally produced borrelia-specific antibodies, or rarely amplification of *Borrelia burgdorferi* sensu lato (Bbsl) DNA from the CSF sample. LA appears as mono-/oligoarthritis and borrelia-specific IgG antibodies confirm the clinical diagnosis. Sometimes the joint involvement might be indicated by amplifying Bbsl DNA from a synovial fluid sample. The Finnish criteria are consistent with the recommendations in the European Concerted Action on Lyme Borreliosis (EUCALB) (*3*).

Results

Demographic Characteristics of LB Case-Patients in Hilmo Register, 1996–2014

The bimodal age distribution was comparable to NIDR with the difference of female gender being clearly overrepresented in all age-groups apart from the oldest (over 70 years; Technical Appendix Figure 2). Hilmo represent LB cases diagnosed and treated in inpatient health care; either in hospitals or outpatient clinics of hospitals. There is a great overlap between Hilmo and NIDR since the LB cases diagnosed in inpatient health care are most likely confirmed by laboratory testing.

Clinical Picture of LB Case-Patients

The clinical picture of the LB cases was only studied using data in Hilmo. Between 1996 and 2014, 968 LNB cases and 450 LA cases were identified in Hilmo. The number of LNB cases was a few dozen yearly with a peak of 129 cases (2.4/100,000 population) in 2011. LNB cases represented 5.4 to 12.2 percent of all LB cases in Hilmo register during 1996–2014. The number of LA cases varied between 10 and 40 during the register period, ranging from 4 to 12.5 percent of the total number. The LA incidence never exceeded 1.0 per 100,000 population. The bimodal age-specific distribution of LNB and LA incidences were seen both in females and males. In LNB, the first peak occurred in the age-group 5–9 years in both sexes and the second peak in 60–69 years of age. In LA, the distribution was otherwise the same but the first peak occurred in the age-group of 10–14 years with both sexes. There were no significant differences in the LNB or LA incidences by HDs.

References

1. Stanek G, Strle F. Lyme borreliosis: a European perspective on diagnosis and clinical management. Curr Opin Infect Dis. 2009;22:450–4. PubMed http://dx.doi.org/10.1097/QCO.0b013e32832ee880

2. Stanek G, Wormser GP, Gray J, Strle F. Lyme borreliosis. Lancet. 2012;379:461–73. PubMed http://dx.doi.org/10.1016/S0140-6736(11)60103-7

3. Huppertz HI, Böhme M, Standaert SM, Karch H, Plotkin SA. Incidence of Lyme borreliosis in the Würzburg region of Germany. Eur J Clin Microbiol Infect Dis. 1999;18:697–703. PubMed http://dx.doi.org/10.1007/s100960050381

Technical Appendix Table 1. Hospital district–specific Lyme borreliosis incidences based on NIDR data, 2010–2014

Hospital district	Incidence, 2010–2014	Incidence rate ratio	95% CI	p value
The Åland Islands	1597.0	1.06	1.06–1.07	<0.05
Southwest Finland	46.3	1.08	1.07–1.09	<0.05
Helsinki and Uusimaa	34.4	1.08	1.08–1.09	<0.05
Satakunta	13.2	1.04	1.02–1.05	<0.05
Tavastia Proper	3.9	1.01	0.99–1.04	0.38
Pirkanmaa	1.5	1.03	0.99–1.06	0.10
Päijät-Häme	10.8	1.09	1.07–1.12	<0.05
Kymenlaakso	56.7	1.12	1.11–1.13	<0.05
South Karelia	48.7	1.12	1.10–1.13	<0.05
Southern Savonia	13.3	1.05	1.02–1.07	<0.05
Eastern Savonia	14.7	1.03	1.00–1.05	<0.05
North Karelia	15.8	1.06	1.05–1.08	<0.05
Northern Savonia	14.2	1.00	0.99–1.02	0.53
Central Finland	7.8	1.05	1.03–1.97	<0.05
Southern Ostrobothnia	1.8	1.02	0.98–1.07	0.30
Vaasa	31.2	1.17	1.14–1.19	<0.05
Central Ostrobothnia	8.7	1.01	0.98–1.04	0.55
Northern Ostrobothnia	1.3	0.99	0.96–1.02	0.47
Kainuu	1.8	1.09	1.00–1.18	0.05
Länsi-Pohja	3.1	1.09	1.02–1.17	<0.05
Lapland	3.9	1.09	1.04–1.14	<0.05

Technical Appendix Table 2. Hospital district–specific Lyme borreliosis incidences based on Avohilmo data, 2010–2014

Hospital district	Incidence, 2010–2014	Incidence rate ratio	95% CI	p value
The Åland Islands	884.6	0.98	0.93–1.04	0.47
Southwest Finland	83.3	1.06	1.01–1.11	<0.05
Helsinki and Uusimaa	63.1	1.24	1.20–1.27	<0.05
Satakunta	28.4	1.14	1.02–1.27	<0.05
Tavastia Proper	17.3	1.02	0.87–1.20	0.79
Pirkanmaa	17.1	1.05	0.96–1.15	0.32
Päijät-Häme	29.9	0.88	0.79–0.98	<0.05
Kymenlaakso	142.9	1.03	0.98–1.09	0.25
South Karelia	161.8	0.91	0.86–0.97	<0.05
Southern Savonia	47.0	1.17	1.03–1.32	<0.05
Eastern Savonia	81.0	1.20	1.04–1.39	<0.05
North Karelia	66.4	1.02	0.94–1.11	0.56
Northern Savonia	47.5	1.19	1.10–1.29	<0.05
Central Finland	49.7	1.22	1.12–1.32	<0.05
Southern Ostrobothnia	18.2	1.29	1.11–1.50	<0.05
Vaasa	40.3	1.09	0.98–1.21	0.13
Central Ostrobothnia	29.4	0.90	0.75–1.08	0.26
Northern Ostrobothnia	4.8	1.22	1.00–1.49	0.06
Kainuu	4.2	1.25	0.76–2.06	0.37
Länsi-Pohja	10.8	0.95	0.68–1.32	0.76
Lapland	5.5	1.28	0.90–1.83	0.16

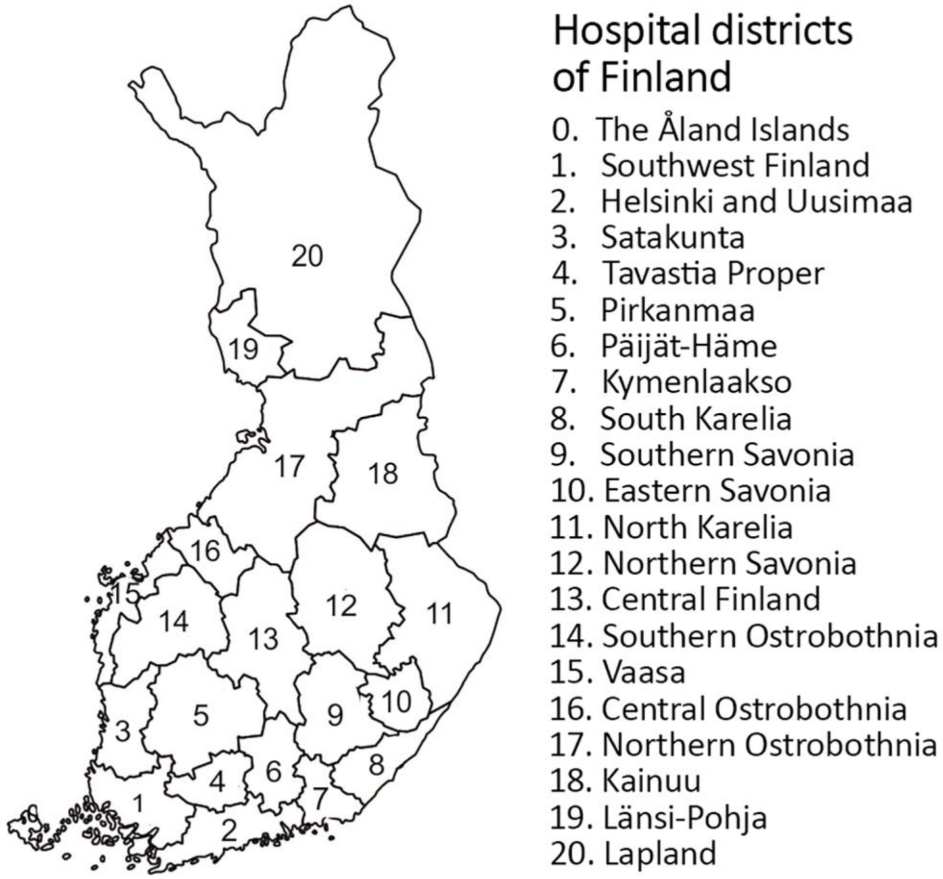

Technical Appendix Figure 1. The hospital districts of Finland.

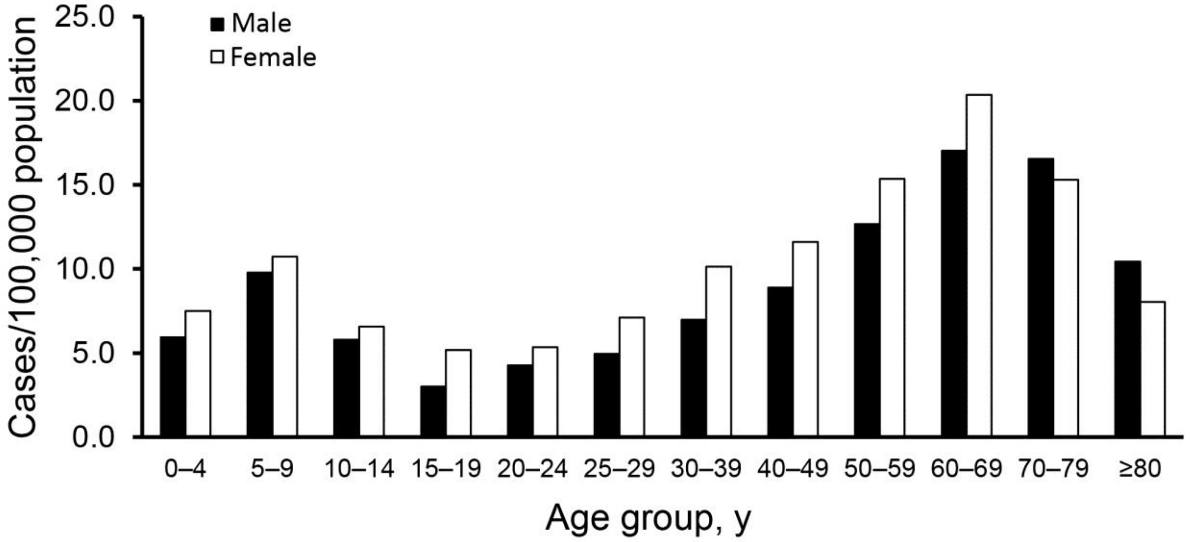

Technical Appendix Figure 2. Incidence rates of hospital discharge (Hilmo)–based Lyme borreliosis cases, by age and sex of case-patients, Finland, 1996–2014.
